# A detailed clinical study of pain in 1957 participants with early/moderate Parkinson's disease

**DOI:** 10.1016/j.parkreldis.2018.06.001

**Published:** 2018-11

**Authors:** Monty A. Silverdale, Christopher Kobylecki, Lewis Kass-Iliyya, Pablo Martinez-Martin, Michael Lawton, Sarah Cotterill, K. Ray Chaudhuri, Huw Morris, Fahd Baig, Nigel Williams, Leon Hubbard, Michele T. Hu, Donald G. Grosset, UK Parkinson's Pain Study Collaboration

**Affiliations:** a Department of Neurology, Salford Royal NHS Foundation Trust, Manchester Academic Health Science Centre, University of Manchester, United Kingdom; b National Center of Epidemiology and CIBERNED, Carlos III Institute of Health, Madrid, Spain; c Dept. of Population Health Sciences, Bristol Medical School, University of Bristol, United Kingdom; d Centre for Biostatistics, School of Health Sciences, University of Manchester, United Kingdom; e Dept. Basic and Clinical Neuroscience, The Maurice Wohl Clinical Neuroscience Institute, King's College London, United Kingdom; f Department of Clinical Neuroscience, UCL, Institute of Neurology, United Kingdom; g Division of Neurology, Nuffield Department of Clinical Neurosciences, Oxford University, United Kingdom; h Division of Psychological Medicine and Clinical Neurosciences, Cardiff University, Cardiff, United Kingdom; i Department of Neurology, Institute of Neurological Sciences, Queen Elizabeth University Hospital, Glasgow, United Kingdom

**Keywords:** Pain, Parkinson's disease, Nonmotor, Central sensitization, Musculoskeletal

## Abstract

**Introduction:**

The causes of pain in early/moderate Parkinson's disease (PD) are not well understood. Although peripheral factors such as rigidity, reduced joint movements and poor posture may contribute towards the development of pain, central mechanisms including altered nociceptive processing may also be involved.

**Methods:**

We performed a large clinical study to investigate potential factors contributing towards pain in early/moderate PD. We recruited 1957 PD participants who had detailed assessments of pain, motor and non-motor symptoms. The King's Parkinson's Pain scale was used to quantify different subtypes of pain.

**Results:**

85% of participants reported pain (42% with moderate to severe pain). Pain influenced quality of life more than motor symptoms in a multiple regression model. Factors predicting overall pain severity included affective symptoms, autonomic symptoms, motor complications, female gender and younger age, but not motor impairment or disease duration. There was negligible correlation between the severity of motor impairment and the severity of musculoskeletal or dystonic pain as well as between the severity of OFF period motor problems and the severity of OFF period pain or OFF period dystonic pain. Features of central sensitization, including allodynia and altered pain sensation were common in this population. The use of drugs targeting central pain was very low.

**Conclusions:**

Pain in early/moderate PD cannot be explained by peripheral factors. Central causes may play a much more important role than previously considered. These results should lead to a major shift in the investigation and management of this common and disabling symptom.

## Highlights

• Pain is common in early/moderate PD and impairs quality of life more than motor symptoms
• No correlation between mobility problems and musculoskeletal or radicular pain in early/moderate PD
• No correlation between OFF motor symptoms and OFF period pain in early/moderate PD
• Features of central pain sensitization are common in early/moderate PD
• Central factors are a more important cause of pain in early/moderate PD than previously considered

## 1 Introduction

Chronic pain affects 60–80% of Parkinson's disease (PD) patients and is often a disabling symptom [[1], [2], [3]]. In the widely used Ford classification, the most common subtypes of PD pain are musculoskeletal, radicular and dystonic [4]. Central pain is thought to be fairly uncommon in PD [1,2,4]. Pain in PD may be a direct consequence of other disease-related symptoms: mobility problems, including stiffness, reduced joint movements and poor posture are felt to be the main cause of musculoskeletal pain and radicular pain; OFF-period mobility problems and dystonic contractions are thought to be the main cause of OFF-period pain [4,5].

While chronic pain can be caused by peripheral tissue damage, an alternative mechanism is dysfunction of pain regulatory systems within the brain, leading to amplification of the pain signal (central sensitization). It is increasingly recognised that many if not most cases of chronic pain are due to altered nociceptive processing within the central nervous system [6,7].

There are several potential mechanisms that may lead to enhanced central processing of pain signals in PD. The dopaminergic pathway from the ventral tegmental area to the nucleus accumbens is an important anti-nociceptive pathway, which degenerates in PD [8]. Furthermore brainstem serotonergic and norepinephrinergic neurons degenerate prior to the onset of motor symptoms in PD [9,10]. Degeneration of these neurons is proposed to be a cause of affective and autonomic symptoms in PD [10]. However these neurons are also the source of powerful ascending and descending anti-nociceptive pathways and their degeneration would therefore be predicted to cause central sensitization of pain signals [11]. Indeed many analgesic drugs, including duloxetine and amitriptyline act through enhancing transmission in these pathways [12]. Reduced pain thresholds [13], altered pain evoked electrical responses [14] and increased pain evoked cortical network activation [15,16], all provide evidence that altered central processing of pain may be an important contributing factor towards pain in PD.

We had several objectives in this study:

1) We hypothesised that pain is an important symptom leading to impaired quality of life in PD. We compared the contribution of pain and motor symptoms in determining quality of life in a large population of participants with relatively early stage PD.
2) We hypothesised that a large detailed clinical study would help us to understand the causes as well as the potential treatments of pain in PD. We used the King's Parkinson's Pain scale to classify and quantify PD pain into different subtypes [17]. We investigated whether the severity of other disease-related symptoms correlated with the severity of the pain subtypes felt to be caused by these symptoms, including whether or not the severity of mobility problems correlated with the severity of musculoskeletal and radicular pain. We also investigated for symptoms suggesting centrally-generated pain including cutaneous allodynia.
3) We hypothesised that pain frequency and severity in PD would correlate with the severity of other symptoms attributable to serotonergic and norepinephrinergic depletion, including anxiety, depression and autonomic dysfunction.

## 2 Methods

### 2.1 Ethical approval

The study was carried out in accordance with the Declaration of Helsinki and authorized by a UK ethics committee (National Research Ethics Service Committee North West – Preston). All patients gave written consent prior to any study procedures.

### 2.2 Participants

Participants were recruited from 68 centres throughout the UK. All participants were recruited from one of two large UK multi-centre longitudinal epidemiological and biomarker studies in PD, those being the Tracking Parkinson's study [18] and the Oxford Monument Discovery Study [19]. The inclusion and exclusion criteria for these other studies have been previously published [18,19]. The pain study was a sub-study of these other studies using the same research nurses, although it was funded and run separately. It was performed at a single occasion at any one of the main study visits.

### 2.3 Pain assessments

Pain was assessed using the Short Form McGill Pain Questionnaire (SFMPQ) [20], Visual Analogue Scale (VAS) for pain severity over the last month and the recently validated Kings Parkinson's Pain Scale (KPPS) [17]. Participants scoring >0 on the SFMPQ were defined as having pain. Similar to previous large pain studies, participants scoring 5 or more on the VAS for pain severity over the last month were defined as having moderate to severe pain [21]. For those with more than 1 type of pain, the most troublesome pain was used to record the SFMPQ and VAS ratings.

The King's Parkinson's Pain Scale quantifies pain across different subtypes. For each subtype the participant rates severity (0–3) and frequency (0–4). The frequency and severity are multiplied together to create a total score for that subtype of pain. The subtypes include musculoskeletal pain (pain around the joints), radicular pain (shooting pain down the limbs), dystonic pain (OFF period pain in a region of dystonia), generalised OFF period pain, lower abdominal pain, visceral pain (pain related to an internal organ such as liver, stomach or bowels), restless leg syndrome (RLS) and central pain (a generalised constant dull aching pain).

### 2.4 Other assessments

Detailed analysis of motor and non-motor symptoms using validated scales was performed to document whether or not the severity of different pain types could be explained by the severity of other motor and non-motor symptoms. The analysis included the Movement Disorder Society Unified Parkinson's Disease Rating Scale (MDS-UPDRS) Part III to document the motor impairment severity, MDS-UPDRS Part IV to document the severity of motor fluctuations, the Leeds Anxiety and Depression Scale (LADS) anxiety score (LADS-A) and depression score (LADS-D) [22] the Scales for Outcomes in Parkinson's disease – Autonomic Symptoms (SCOPA-AUT) to document the severity of autonomic symptoms, the Montreal Cognitive Assessment (MoCA) and the EQ-5D index, a measure of quality of life (QoL).

The Leeds Assessment of Neuropathic Symptoms and Signs (LANSS) scale was used to document the presence of neuropathic pain [23]. The LANSS scale is validated to detect neuropathic pain and uses an assessment of sensory function (cutaneous allodynia and altered pinprick threshold over the painful area) as well as pain descriptors to classify pain which is likely to be centrally generated. Participants were asked whether the pain was better in the ON state, the OFF state or no different. They were also asked which if any of their ‘non Parkinson's medications' improved pain. However we did not collect detailed information regarding which pain medications were being taken at the time of assessment.

All scales were performed in at least 1600 of the participants with the exception of the SCOPA-AUT, which was not part of the Oxford Monument Discovery study therefore was only performed in 816 participants. All scales were performed within 6 months of each other. Thus the motor and non-motor assessments (MDS-UPDRS, LADS, EQ-5D, SCOPA and MoCA) were all done at the same time. The pain assessments (SFMPQ, VAS, LANSS and KPPS) were all done at the same time. In 917 participants the pain assessments were done at the same time as the motor and non-motor assessments. In 527 the pain assessments were done within 6 months after the motor and non-motor assessments. In 513 participants, the pain assessments were done within 6 months before the motor and non-motor assessments. All scales and assessments were performed in the ON state. In the Tracking Parkinson's and Oxford Discovery studies, all participants had the MDS-UPDRS III performed in the OFF state (after 12 h withdrawal of anti-parkinsonian medication) at a single visit. However in many cases the pain assessments were done more than 6 months from the MDS-UPDRS-III OFF so we included MDS-UPDRS-III OFF data only in the 491 participants in whom MDS-UPDRS-III OFF was done within 6 months of the pain assessments.

### 2.5 Statistical analysis

Statistical analysis was performed using SPSS (version 22, IBM). Multiple linear regression analysis was used to investigate factors predicting quality of life (EQ-5D index) in PD. Predictor variables in the model included severity of pain (SFMPQ), severity of motor impairment (MDS-UPDRS-III) and severity of motor complications (MDS-UPDRS-IV).

Multiple linear regression analysis was used to investigate factors predicting the overall severity of pain (SFMPQ) in PD. Candidate variables in the model were MDS-UPDRS-III, MDS-UPDRS-IV, SCOPA-AUT, LADS, MoCA, age, disease duration and female gender.

Correlation analysis was used to investigate the relationship between different types of pain (as measured with the Kings Parkinson's Pain Scale) and other disease related factors. Due to the non-parametric nature of the individual scales, in particular those in the King's Parkinson's Pain Scale, non-parametric (Spearman's rank) correlation analysis was used.

## 3 Results

### 3.1 Study population and pain characteristics

1957 participants were recruited into the UK Parkinson's Pain Study. 65% were male. Table 1 shows the characteristics of the study population. This was a population with relatively early PD. 1648 out of 1944 participants recording SFMPQ (85%) reported pain at the time of assessment. 808 participants (42%) reported moderate to severe pain. The main pain subtypes were: musculoskeletal pain (66% of those with pain), radicular pain (34%), dystonic pain (16%), generalised OFF pain (9%), dyskinetic pain (10%), lower abdominal pain (16%), visceral pain (16%), central pain (27%) and restless leg syndrome (23%). 395 participants reported 1 type of pain, 389 reported 2 types, 281 reported 3 types and the rest reported 4 or more types of pain.

**Table 1:** Characteristics of the study population.

	Mean	SD	n
Age (Year)	68	9.5	1914
Disease Duration (Years)	3.0	2.1	1887
LADS-Anxiety	4.1	3.6	1737
LADS-Depression	4.5	3.2	1729
MDS-UPDRS-III	27	13.6	1657
MDS-UPDRS-III (OFF)	33.5	15.5	495
MDS-UPDRS-IV	1.2	2.4	1685
SCOPA-AUT	12.5	7.2	823
MoCA	25.1	3.6	1706
SFMPQ	6.8	6.6	1944
EQ-5D Index	0.71	0.24	1751

SD, standard deviation; LADS-Anxiety, Leeds Anxiety and Depression Scale-Anxiety; LADS-Depression, Leeds Anxiety and Depression Scale-Depression; MDS-UPDRS-III, Unified Parkinson's disease Rating Scale Part III (motor examination); MDS-UPDRS-IV, Unified Parkinson's disease Rating Scale Part IV (motor complications); SCOPA-AUT, the Scales for Outcomes in Parkinson's disease – Autonomic Symptoms. MoCA, Montreal Cognitive Assessment; SFMPQ, Short Form McGill Pain Questionnaire; EQ-5D Index, Quality of Life Scale.

### 3.2 Effects of pain and motor symptoms on quality of life

Predictors of quality of life (EQ-5D index) in the regression model (Table 2) were motor examination (MDS-UPDRS III), motor complications (MDS-UPDRS IV) and pain (SFMPQ). The model accounted for 26% of the variance in quality of life. In this population of participants with fairly early PD, all of the variables had an effect on quality of life and the effect of pain was higher than that of motor disability or motor fluctuations.

**Table 2:** Multiple linear regression for health-related Quality of life (EQ-5D Index).

	R^2^	Beta	T	95% Confidence intervals		sig
				Lower	upper	
	0.26					
MDS-UPDRS-III		−0.003	−8.4	−0.004	−0.003	< **0.001**
MDS-UPDRS-IV		−0.016	−7.1	−0.021	−0.012	< **0.001**
SFMPQ		−0.014	−16.6	−0.015	−0.012	< **0.001**

MDS-UPDRS-III, Unified Parkinson's disease Rating Scale Part III (motor examination); MDS-UPDRS-IV, Unified Parkinson's disease Rating Scale Part IV (motor complications); SFMPQ, Short Form McGill Pain Questionnaire.

### 3.3 Central mechanisms and pain

33% of those with pain had evidence for altered sensory processing over the painful area, this being either cutaneous allodynia, altered pinprick threshold or both. 10% of participants with pain had a LANSS score of ≥12 indicating the presence of neuropathic pain [23].

### 3.4 Factors predicting pain in PD

Table 3 shows the results of a regression analysis using the overall severity of pain (SFMPQ) as the outcome variable and the severity of other disease related symptoms as predictor variables. The model explained 21% of the overall variance in pain severity. Factors having a statistically significant effect on pain severity were motor complications, autonomic symptoms, affective symptoms, younger age and female gender. The severity of motor impairment and the severity of cognitive impairment did not have a statistically significant effect on the severity of pain.

**Table 3:** Multiple linear regression for pain severity - Short form McGill Pain Questionnaire (SFMPQ).

	R^2^	Beta	t	95% Confidence intervals		sig
				lower	upper	
	0.21					
MDS-UPDRS-III		0.003	0.185	−0.031	0.038	0.853
MDS-UPDRS-IV		0.213	2.121	0.016	0.411	**0.034**
SCOPA		0.224	6.031	0.151	0.297	< **0.001**
LADS		0.224	5.347	0.142	0.307	< **0.001**
MoCA		−0.005	−0.076	−0.135	0.125	0.940
Age		−0.072	−2.801	−0.123	−0.022	**0.005**
Disease Duration		0.071	0.676	−0.136	0.278	0.394
Gender (Female)		1.619	3.450	0.698	2.540	**0.001**

MDS-UPDRS-III, Unified Parkinson's disease Rating Scale Part III (motor examination); MDS-UPDRS-IV, Unified Parkinson's disease Rating Scale Part IV (motor complications); SCOPA-AUT, the Scales for Outcomes in Parkinson's disease – Autonomic Symptoms; LADS, Leeds Anxiety and Depression Scale; MoCA, Montreal Cognitive Assessment.

### 3.5 Medications and pain in PD

Pain was reported to be worse in the OFF state by 17% of participants, whereas 2% felt it was worse in the ON state and 81% felt there was no difference between the ON and OFF state with regards to pain severity. Regarding medications which were being used to treat pain and which were felt by the participant to be effective for PD pain, 28% of those with pain reported benefit from paracetamol (acetaminophen), compared to 12% from non steroidal anti-inflammatory drugs (NSAIDS) and 10% from using opioids. Only 3% of participants with pain derived benefit from drugs targeting central pain mechanisms, including gabapentin, pregabalin, duloxetine and amitriptyline.

### 3.6 Correlation between individual subtypes of pain and other disease related symptoms

Table 4 shows the correlation between different types of pain (as measured with the King's Parkinson's pain scale) and other disease-related symptoms. There was negligible correlation between the severity of musculoskeletal pain and the severity of motor symptoms (MDS-UPDRS III) in both the ON and OFF states. There was weak correlation between the severity of musculoskeletal pain and the severity of motor complications (MDS-UPDRS-IV), autonomic symptoms (SCOPA-AUT), anxiety (LADS-A) and depression (LADS-D).

**Table 4:** Correlation between the severity of individual subtypes of pain and other disease related symptoms.

	Musculoskeletal	Radicular	OFF dystonic	General OFF	Dyskinetic	Lower Abdo	Visceral
MDS-UPDRS-III	0.03	0.05	0.02	0.05^∗^	−0.01	0.04	0.05^∗^
MDS-UPDRS-III (OFF)	0.04	0.08	0.11^∗^	0.06	0.06	0.09^∗^	0.07
UPDRS-IV	**0.11**^∗∗^	**0.12**^∗∗^	**0.27**^∗∗^	**0.20**^∗∗^	**0.15**^∗∗^	0.05^∗^	0.05^∗^
LADS-A	**0.2**^∗∗^	**0.22**^∗∗^	**0.17**^∗∗^	**0.19**^∗∗^	**0.17**^∗∗^	**0.14**^∗∗^	**0.13**^∗∗^
LADS-D	**0.16**^∗∗^	**0.18**^∗∗^	**0.16**^∗∗^	**0.17**^∗∗^	**0.11**^∗∗^	**0.14**^∗∗^	**0.12**^∗∗^
SCOPA-AUT	**0.17**^∗∗^	**0.14**^∗∗^	**0.13**^∗∗^	**0.17**^∗∗^	0.12^∗^	**0.19**^∗∗^	**0.24**^∗∗^
Constipation	**0.10**^∗∗^	0.06^∗^	0.05^∗^	**0.09**^∗∗^	0.04	**0.17**^∗∗^	**0.16**^∗∗^

Spearman's rank correlation coefficient (rho); ^∗^P < 0.05; ^**^P < **0.001**.

Individual pain subtypes were quantified using the King's Parkinson's Pain Scale.

MDS-UPDRS-III, Unified Parkinson's disease Rating Scale Part III (motor examination); MDS-UPDRS-III (OFF), UPDRS-III after 12 h medication withdrawal; MDS-UPDRS-IV, Unified Parkinson's disease Rating Scale Part IV (motor complications); SCOPA-AUT, the Scales for Outcomes in Parkinson's disease – Autonomic Symptoms; LADS-A, Leeds Anxiety and Depression Scale-Anxiety; LADS-D, Leeds Anxiety and Depression Scale-Depression; Constipation, UPDRS 1.11.

Similarly there was negligible correlation between radicular pain severity and the severity of motor symptoms. There was only weak correlation with the severity of motor complications, autonomic symptoms, anxiety and depression.

There was poor correlation between the severity of OFF period motor problems as measured with MDS-UPDRS-III OFF and the severity of either dystonic OFF pain or generalised OFF pain. OFF period pain was significantly correlated with MDS-UPDRS-IV but the correlation was only weak. Dyskinetic pain correlated with the severity of motor fluctuations, although only with a very weak association. Both lower abdominal pain and visceral pain correlated with constipation scores (Q1.11 in MDS-UPDRS). However the association was only weak. Both of these types of pain correlated with autonomic symptoms, again only with a weak association.

## 4 Discussion

### 4.1 Influence of pain on quality of life in PD

Our first hypothesis was that pain is an important symptom leading to impaired quality of life in PD. In our study, pain was extremely common in this large population of participants at a relatively early stage of PD. 85% of participants reported pain and 42% of participants reported moderate to severe pain. Although we did not have a control group, the frequency of pain is clearly much higher than previously reported in the general population. A European study of 46,394 participants, using similar inclusion criteria and definitions of pain severity to our study, noted a prevalence of moderate to severe pain of 19%, although the figure was 13% if only the 3800 UK participants were included [21]. Our results therefore confirm previous studies indicating a very high prevalence of pain in PD [1,2].

In our study pain had an effect on quality of life, which was greater than motor impairment and comparable to the effect of motor complications. Other smaller studies have reported similar findings [24]. Given the amount of research that has gone into understanding mobility problems in PD, it is clear that there needs to be more research into understanding the causes of pain in PD with a view to developing improved treatments.

It should be highlighted that participants in our study had fairly mild motor impairment/complications and results may be different in a more severely-affected population.

### 4.2 Relationship between pain and other disease symptoms

Our second hypothesis was that a large and detailed clinical study would help us to understand the causes and therefore identify potential treatments for pain in PD. The severity of motor impairment did not predict the overall severity of pain (SFMPQ). Furthermore the severity of motor fluctuations only weakly predicted variance in the severity of pain. Thus mobility issues are probably not a very important cause for the markedly increased frequency and severity of pain in PD.

The size and scope of our study enabled us for the first time, to perform an analysis of the factors which may be contributing towards different subtypes of pain in PD. Musculoskeletal pain and radicular pain are the most common types of pain in PD. We saw negligible correlation between these pain subtypes and MDS-UPDRS-III, suggesting that mobility factors are not an important cause. Off period dystonic pain is common in PD and is understandably attributed to painful dystonic muscle contractions despite the fact that with one or two exceptions, dystonia is usually not a painful condition. It is very interesting therefore that there was negligible correlation between the severity of dystonic pain and the severity of the MDS-UPDRS-III OFF motor scores. Similarly there was negligible correlation between generalised OFF period pain and MDS-UPDRS-OFF scores. Dyskinetic pain was only weakly correlated with motor fluctuation scores (MDS-UPDRS-IV). Abdominal pain and visceral pain correlated with constipation severity but with only a small effect size.

Our results suggest that peripheral factors are not an important cause of these different subtypes of pain in PD. Although we attempted to analyse peripheral factors in detail, we cannot exclude the possibility that others which could have contributed to pain were not adequately assessed in our study. However the high prevalence of either cutaneous allodynia or altered pinprick threshold over the painful area suggests that central mechanisms may be a more important contributing factor towards pain than previously considered. Indeed the LANSS scale classified 10% of participants as having centrally-generated neuropathic pain. It is well recognised that there is a very poor correlation between the severity of osteoarthritic problems on imaging and pain severity [25]. In PD patients with what is described to be musculoskeletal shoulder problems, there is enhanced sensory processing over the painful shoulder implicating a role for central factors in contributing towards musculoskeletal pain in PD [26].

Despite the lack of detailed data on analgesic use, it is noteworthy that only a very small proportion of participants were using drugs which target central mechanisms. Our results suggest that more widespread use of these medications should be considered as a treatment for pain in PD.

Our study data confirms previous reports that female sex is a contributing factor towards pain in PD [1]. Interestingly, age was inversely associated with pain severity. The relationship between pain and age is complex, although similar findings have previously been reported in the general population [27]. Pain due to PD has been linked to younger age and age at onset [2]. Pain was not associated with disease duration in our study, although the predominance of fairly early PD participants in our study precludes too much importance being given to this finding.

We acknowledge study limitations which could have affected these correlations. The population was of early/moderate PD and results may be different in more severely-affected participants. There was a delay of up to 6 months between pain and motor assessments which could have affected correlations. We did not assess in detail the potential confounding effects of analgesic use.

### 4.3 Relationship between pain and symptoms of brainstem monoamine dysfunction

Our third hypothesis was that pain frequency and severity in PD would correlate with the severity of other symptoms attributable to serotonergic and norepinephrinergic depletion. Consistent with this, the severity of autonomic symptoms, anxiety and depression predicted the severity of pain. A recent study showed no association between pain severity and the severity of small fibre neuropathy in PD [28], suggesting that central factors may be more important. Although our data do not enable us to establish a definite cause or direction of this association, we propose that a shared pathophysiology of degeneration in brainstem monoamine pathways from premotor stages of PD (Braak stage 2) onwards, contributes to causing autonomic symptoms, affective symptoms and altered processing of pain in PD.

The results of this study suggest that a major shift in the way we approach this common and disabling symptom in PD is required. In one small open study, central pain in PD was reduced by duloxetine [29], and further studies with this type of medication are clearly warranted, and should assess effects on the various pain subtypes. Similarly, deep brain stimulation improved pain in PD, separate from the improvement in mobility symptoms [30], again stressing the role of central mechanisms, distinct from those driving motor impairment.

### 4.4 Limitations of the study

We acknowledge that there are several limitations to our study which mean that the results must be interpreted cautiously. The study population was mainly of early and moderate cases and detailed data on other comorbidities and analgesic use were not available, so the results are not necessarily generalisable to all patients with PD. The LANNS scale while not specifically validated in PD is rated as ‘suggested’ by experts in PD related pain [31]. Although participants were assessed ON medication, we did not standardise the timing of assessments with regards to ON/OFF state and in some cases the pain assessments were not performed at the same time as other study measures. As with any large study there is some missing data which could have affected the results.

We acknowledge that it is possible our results are caused in part by the type of patients enrolled and the limitations of our methodology. Other similar studies with different populations and methodologies will help to clarify this point.

## 5 Conclusions

The UK Parkinson's Pain Study is the largest and most detailed study of pain in PD ever performed. Detailed phenotyping and high power from a large number of participants allowed us for the first time to explore in detail the factors contributing to subtypes of pain in PD. In our study population, we found that peripheral factors are not an important cause of pain in PD, and conclude that central factors are more important than previously considered.

## Principal investigators

C.Borland, A. Graham, S. Guptha, P. Worth, P. Sarda, W. Dwornik, G. Mamutse, J. Hindle, A. Church, R. Athey, D. Bathgate, N. Pavese, U. Nath, M. Carson, R. Walker, T. Foltynie, A. Misbahuddin, A. Schrag, S. Sveinbjornsdottir, Y. Adenwala, B. Boothman, J. George, M. O'Neill, J. Raw, M. Steiger, M. Wilson, K. Amar, S. Arianayagam, Z. Hemsley, H. Roberts, J. Stern, T. Andrews, D. Paviour, J. Frost, C. Carroll, V. Lyell, T. Harrower, R. Sheridan, P. Critchley, L. Sugathapala, J. Sharma, C. Clarke, S. Ellis, K. Nithi, N. Kock, Z. Dhakam, C. Ruffman, P. Tomlinson, T. Quinell, S. Molloy, A. Whone, O. Bandmann, E. Capps, D. Burn, J. George, R. Bland, N. Bajaj, T. Ward.

## Research nurses

C.Downes, C. Vandor, J. Frost, D. Cooper, K. Dixon, R. Norton, M. Clarke, C. Hall, O. Olanrewaju, V. Hetherington, K. Blachford, S. Guptha, B. Bishop, D. Nesbitt, J. McEntee, F. Frenais, A. Hursey, E. Visentin, C. Edwards, J. Newman, r. James, E. Johnson, K. Ullyart, J. Brooke, I. Bataller, J. Rickett, T. Mahan, L. Craven, K. Powell, R. Humphries, B. Wilson, M. Trimmer, L. Whelan, A. McNichol, T. Fuller, L. Alderton, A. Henderson, P. Brown, V. Visentin, C. Parker, S. Large, H. Vanek, D. Wilson, A. Misbahuddin, F. Foster, S. Dube, E. Gunter, K. Amar, S. Dodds, G. Brown, G. Webster, P. Critchley, L. Foster, L. Dudgeon, K. Holmes, D. Nithi, K.K.Nithi, A. Donaldson, A. Dougherty, M. Misbahuddin, S. Atkinson, G. Carey, L. Catterall, D. Dellafera, C. Sunderland, A. Lyle, C. Sequeira, M. Hare, E. Ekins, K. Maitland, C.O'Reilly, M. Prabhakaran, P. Paterson, J. Connell, A. Pilcher, J. Birt, N. Vernon, T. McElwaine, M. Humphries, A. Lehn, T. Murphy, D. Critchley, M. Rolinsky, N. Temple, C. Brugaletta, S. Cable, D. Mills, S. Levy, E. Templeman, L. Wyatt, I. Massey, R. Innis, C. Ruffman, C. Schofield, E. Oughton, A. Davies, A. Lehn, M. Korley, N. Verstraelen, S. Morgan, W. Dwornik, J. Gilford, P. Aruldoss, A. Foster, C. Chaudhuri, L. Gethin, Y. Croucher, M. Williams, T. Roberts, R. Carson, R. Athey, V. Agarwal, P. Rachman, V. Ludley, T. Talbot, I. Inniss, R. Gentle, C. Hewitt, J. Stickley, E. White, K. Ward, S. Butler, S. Gallahawk, R. Fernandes, P. Zettergren, B. Tobin, K. Huyalt, U. Ullyart, E. Henderson, P. Forbes, M. Heywood, J. O'Brien, R. Rettergra, F. Bowring, S. Large.

## Author roles

Monty A Silverdale: 1A, 1B, 1C, 2A, 2B, 3A.

Christopher Kobylecki. 1B, 1C, 2C, 3B.

Lewis Kass-Iliyya. 1B, 1C, 2C, 3B.

Pablo Martinez-Martin. 2C, 3B.

Donald G Grosset. 1A, 1B, 1C, 3B.

Michele T. Hu. 1B, 1C, 3B.

Michael Lawton. 2C, 3B.

Sarah Cotterill. 2C, 3B.

K. Ray Chaudhuri. 1C, 3B.

Huw Morris. 1C, 3B.

Fahd Baig. 1C, 3B.

Nigel Williams. 1C, 3B.

Leon Hubbard. 1C, 3B.

1 Research project: A. Conception, B. Organization, C. Execution;

2 Statistical Analysis: A. Design, B. Execution, C. Review and Critique;

3 Manuscript: A. Writing of the first draft, B. Review and Critique.

## Financial disclosures for all authors (for the preceding 12 months)

Monty Silverdale has received grants from Michael J. Fox Foundation, Parkinson's UK, NIHR and the Dystonia society.

Christopher Kobylecki has received grants from Michael J. Fox Foundation and the Dystonia Society, Honoraria from UCB, Ipsen, Allergan and Britannia.

Lewis Kass-Iliyya has no financial disclosures.

Pablo Martinez-Martin has received honoraria from Editorial Viguera, International Parkinson and Movement Disorder Society and HM Hospitales de Madrid, License fee payments for the King's Parkinson's Disease Pain scale and a Grant from the International Parkinson and Movement Disorder Society for attending the Congress of the Society 2017.

Donald Grosset has received grant funding from the Neurosciences Foundation, Michael's Movers, and Parkinson's UK, and honoraria from Bial Pharmaceuticals and GE Healthcare.

Michele Hu has received grants from Parkinson's UK, Oxford BRC.

Michael Lawton has no financial disclosures.

Sarah Cotterill has no financial disclosures.

K Ray Chaudhuri has received honoraria from AbbVie, Britannia, UCB, Mundipharma, Zambon, GKC, Bial, Grants from EU, Parkinson’s UK, NIHR, PDNMG, Kirby Laing, NPF, Consultancies from Britannia, AbbVie, Neuronova, UCB, Advisory Boards from AbbVie, UCB, Sunovion, Pfizer, Jazz Pharma, GKC, Bial and Intellectual Property rights for KPPS and PDSS-2.

Huw Morris has received honoraria from E-Scape, Bristol-Myers-Squibb, Wellcome Trust and Lecture fees UCB Pharma, Wellcome Trust.

Fahd Baig has no financial disclosures.

Nigel Williams has no financial disclosures.

Leon Hubbard has no financial disclosures.

## Financial disclosures/conflicts of interest relevant to manuscript

None.

## Funding sources of the study

This work was funded by Parkinson's UK (grant number K1301). The funding source had no other involvement in the study.
